# Focusing on the long‐term recovery of severe acute respiratory syndrome coronavirus 2 infection: Clinically relevant observations

**DOI:** 10.1002/ctd2.99

**Published:** 2022-06-22

**Authors:** Chunxia Wang, Yucai Zhang

**Affiliations:** ^1^ Department of Critical Care Medicine Shanghai Children's Hospital Shanghai Jiao Tong University School of Medicine Shanghai China; ^2^ Institute of Pediatric Infection Immunity, and Critical Care Medicine Shanghai Jiao Tong University School of Medicine Shanghai China; ^3^ Institute of Pediatric Critical Care Shanghai Jiao Tong University Shanghai China

**Keywords:** impaired lung function, indicators, long‐term recovery, lung fibrosis, SARS‐CoV‐2 infection

## Abstract

**Background:**

The long‐term implications of COVID‐19 attract global attention in the post‐COVID‐19 pandemic era. Impaired lung function is the main sequelae in adults' survivors of SARS‐CoV‐2 infection.

**Methods and Results:**

The plasma proteomic pattern provides novel evidence on multiple biological domains relevant to monitoring lung function and targeting the clinical application in adults with acute respiratory distress syndrome (ARDS) secondary to SARS‐CoV‐2 infection (SARS‐CoV‐2‐ARDS). Preliminary studies support the evidence of pulmonary function tests (PFT) and computed tomography (CT) scan as routine follow‐up tools. Combining the early fibrotic indicators and D‐dimer levels could prove the validity and reliability of the proactive management of lung function assessment during the long‐term recovery in SARS‐CoV‐2 infection.

**Conclusion:**

In summary, protocolized PFT and CT scan and effective biomarkers for early fibrotic changes should be applied to clinical practice during the long follow‐up in patients with severe COVID‐19.

## 1 COMMENTARY

The possible long‐term health sequelae of severe acute respiratory syndrome coronavirus 2 (SARS‐CoV‐2) infection have become a global concern.^1^ García‐Hidalgo MC et al^2^ first reported the plasma proteomic profiling in adults after 3‐month of SARS‐CoV‐2 infection to reveal the underlying mechanisms of lung diffusion impairment (defined as carbon monoxide diffusing capacity [DLCO] < 60%) in Clinical and Translational Medicine. The authors concluded that 1) At the 3‐month follow‐up, 30% of patients presented moderate‐to‐severe pulmonary diffusion impairment; 2) The analysis identified a signature of 35 proteins implicated multiple biological pathways including lung epithelial, endothelial and immune cells after adjusted by age, sex, previous chronic pulmonary disease, smoking history, glomerular filtration and the use of corticoids after hospital discharge; 3) No association was observed between blood viral load and diffusion impairment.

These findings are appreciable for constituting a source of targets for therapeutic strategies. However, only four panels including organ damage, immune response, inflammation and metabolism were analyzed, which still needs more comprehensive analysis. Candidates’ targets should be further confirmed in a validation cohort. In this commentary, we would culminate an insight into the incidence, risk factors, and biomarkers for monitoring the impaired lung function during the long‐term recovery of patients with SARS‐CoV‐2 infection.

## 2 THE METHODS FOR MONITORING LUNG FUNCTION

The main tools for monitoring lung function in coronavirus disease 2019 (COVID‐19) survivors after hospital discharge include pulmonary function tests (PFT), computed tomography (CT), and laboratory variables. The most common PFT abnormalities were impaired DLCO and alveolar volume, reduced DLCO and/or reduced forced vital capacity.^3^, ^4^ A combination of ground‐glass opacity (GGO) and the reticular pattern was the most common CT finding. GGO mostly showed a faint appearance. Bronchial dilatation and architectural distortion coexisted in most patients. The consolidation pattern only showed in a few cases. CT abnormalities were mostly bilateral in 97% of cases without any zonal predominance.^4^ Besides, higher D‐dimer, matrix metalloproteinase 7 (MMP7), and periostin levels were found in patients with CT abnormalities,^4^, ^5^ and high MMP7, MMP1, and periostin levels were associated with early fibrotic changes.^5^ Up to date, there is still no consensus about the procedural clinical and laboratory indexes for the long‐term follow‐up examination in patients with SARS‐CoV‐2 infection.

## 3 THE INCIDENCE OF IMPAIRED LUNG FUNCTION

At 6 months after acute infection, alteration of DLCO still remained in 56% of patients who required high‐flow nasal cannula or invasive mechanical ventilation and 29% of those who required supplemental oxygen.^6^ The proportion of patients with DLCO < 80% was 54.6% and 47% at 60 and 180 days after acute infection in patients with moderate and severe COVID‐19 pneumonia, and 38% of those patients might undergo early fibrotic lesions.^5^ Consistently, 52% of COVID‐19 patients that required intensive care showed reduced DLCO at 3–6 months post discharge.^3^ In another aspect, for patients with cancer who are scheduled to resume anti‐cancer treatment after recovering from COVID‐19, the changes of pulmonary functional during and after administering chemotherapy is linked to the loss of DLCO during follow‐up period.^7^ According to the results of CT scan, 81% (74/91) of patients had CT abnormalities combined GGO and reticular pattern (62%, 46/74) along with architectural distortion (92%, 68/74) and bronchial dilatation (89%, 66/74) at a median of 105 days from symptom onset in survivors of SARS‐CoV‐2 infection‐induced acute respiratory distress syndrome (ARDS).^4^ These studies amplified the importance about the assessment of DLCO and CT scan in severe COVID‐19 patients.

## 4 THE RISK FACTOR FOR LONG‐TERM IMPAIRED LUNG FUNCTION

The risk factors for long‐term impaired lung function have not been fully defined. In COVID‐19 without intensive care, male, older, and who received steroids during the hospital stay were associated with PFT or CT abnormalities at follow‐up.^4^ Moreover, risk factors for impaired DLCO include sex, age (above 60 years), peak radiographic assessment of lung oedema score, need for mechanical ventilation and longer ICU stay, as well as lower levels of C‐reactive protein, the nadir of blood lymphocytes and leukocytes at admission.^3^ In addition, early fibrotic changes were associated with higher levels of MMP7, MMP1, and periostin in patients with moderate and severe COVID‐19 pneumonia.^5^ Given that the basal circulating interferon‐γ, transforming growth factor‐β1, and connective tissue growth factor levels were correlated to ARDS‐induced lung fibrosis, the potential value of warning lung fibrosis needs further study in COVID‐19. A previous study indicated that a high D‐dimer level was an indicator for early anticoagulation therapy in COVID‐19 patients. However, whether the D‐dimer level is an effective indicator for monitoring the long‐term recovery of patients with COVID‐19 still is controversial.

## 5 THE LONG‐TERM IMPAIRED ORGAN FUNCTION IN CHILDREN WITH SARS‐CoV‐2 INFECTION

Compared with adults, pulmonary function is rarely impaired in children and adolescents. Only 5.3%–14% of children presented abnormal PFTs, and impaired diffusing capacity was found in 30% of patients infected with SARS‐CoV‐2.^8^, ^9^ In contrast to rarely impaired pulmonary function, respiratory system symptoms persist in approximately 30% of patients at 3 months after SARS‐CoV‐2 infection.^9^ Compared with adults, severe hepatitis could be a critical concern for a long‐term follow‐up in children with COVID‐19.^10^ Consistently, Brodin et al. hypothesized that severe acute hepatitis in children was possibly related to a SARS‐CoV‐2 superantigen mechanism without a clear conclusion until now.

## CONFLICT OF INTEREST

The authors declare that they have no conflict of interest.
